# Implications of segmental and lobar tracheobronchial anomalies in congenital heart disease: a 12-year retrospective CT analysis

**DOI:** 10.3389/fradi.2025.1697305

**Published:** 2026-03-04

**Authors:** Mohamad Yanuar Amal, Shyh-Jye Chen

**Affiliations:** 1 Division of Pediatric Radiology, Department of Radiology, Dr. Cipto Mangunkusumo General Hospital, Faculty of Medicine Universitas Indonesia, Jakarta, Indonesia; 2 Department of Medical Imaging, National Taiwan University Hospital, Taipei, Taiwan

**Keywords:** tracheobronchial anomalies, congenital heart disease (CHD), pulmonary atresia, persistent left superior vena cava (PLSVC), computed tomography (CT)

## Abstract

**Objectives:**

This study aims to evaluate the prevalence and anatomical patterns of tracheobronchial anomalies and to analyze their associations with different types of congenital heart disease (CHD) using retrospective CT data.

**Introduction:**

Tracheobronchial anomalies, including variations in the branching patterns of the trachea and bronchi, are relatively rare but clinically significant. These anomalies, which are often associated with CHD, can complicate respiratory function and airway management. Despite their low prevalence, early identification and understanding of these anatomical variations are essential to improve patient outcomes.

**Methods:**

This retrospective study was conducted at the National Taiwan University Hospital and included patients who underwent computed tomography (CT) imaging between December 2012 and February 2024. The inclusion criteria were strictly defined to focus on patients diagnosed with a lobar or segmental tracheal bronchus anomaly, as identified on their initial CT scan.

**Results:**

Among 356 patients diagnosed with tracheobronchial anomalies, 223 had concurrent CHD. The study found a significant association between tracheobronchial anomalies and CHD, particularly in conditions such as a right-sided aortic arch and pulmonary atresia. In addition, the presence of persistent left superior vena cava was notably higher in patients with CHD.

**Discussion:**

The study accentuates the importance of early detection of tracheal bronchus anomalies, particularly in patients with CHD. Early detection through advanced imaging techniques is critical for minimizing complications. This study supports the “space availability” hypothesis of embryological development of these anomalies. Moreover, the significant association between tracheal bronchus and conditions such as pulmonary atresia and a right-sided aortic arch suggests a shared embryological pathway.

**Conclusion:**

Tracheobronchial anomalies are clinically significant, particularly in the context of CHD. Timely identification, coupled with a multidisciplinary approach, is crucial to enhancing patient outcomes and reducing complications. Future research should focus on the genetic and environmental factors that contribute to the development of these conditions.

## Introduction

Medical literature has documented several notable irregularities in bronchial branching patterns, with an estimated prevalence ranging from 0.1% to 2% in the general population. These variations in bronchial anatomy may present as congenital anomalies affecting the trachea, main bronchi, and intermediate bronchus (1). Despite the potential significance of these anomalies, routine chest computed tomography (CT) scans are typically capable of detecting such deviations. However, such anomalies are not uncommon and are frequently disregarded or overlooked during initial assessments, highlighting the importance of thorough examination and careful interpretation of imaging findings in clinical practice (2).

Branching patterns in tracheobronchial anomalies vary considerably. Some anomalies, such as bronchial situs anomalies, upper lobe tracheobronchial branching anomalies, and bridging bronchi, are commonly associated with congenital heart disease (CHD), lung abnormalities, or foregut malformations. This complexity reinforces the need for comprehensive evaluation and management (3).

The first documented description of a tracheal bronchus originating from the trachea was credited to Sandifort in 1785. Ming and Lin noted a 13-fold increase in the prevalence of right tracheal bronchi among children with congenital heart disease. The medial or apical segmental bronchus of the right upper lobe typically arises from the right wall of the trachea. However, the occurrence of the right upper lobe bronchus, along with its segmental and sub-segmental branches, originating directly from the trachea, is exceedingly rare, with a reported incidence of only 0.2% (1).

The hypotheses regarding the pathogenesis of a tracheal bronchus are based on previous research. These theories shed light on its embryological origins and potential underlying mechanisms.

Bremer's hypothesis proposes that the failure of regression of tracheal buds *in utero* provides a compelling explanation for the development of a tracheal bronchus. The higher incidence of aberrant tracheal buds in human embryos compared to the general population suggests that this developmental process may be significant in understanding the origins of a tracheal bronchus. The theory that a tracheal bronchus arises from a disruption in normal embryogenesis emphasizes the complexity and precision of the processes involved in developing the respiratory system (4). This perspective illustrates the intricate interplay of genetic and environmental factors that govern the formation of anatomical variations such as a tracheal bronchus. Experimental work by Alescio and Cassini, demonstrating tracheal bud induction via bronchial mesenchyme transplantation, provides valuable insight into developmental influences. This research suggests that interactions between different tissues during embryonic development may contribute to the formation of a tracheal bronchus, further emphasizing the multifaceted nature of developmental biology (5). Researchers aim to deepen our understanding of the intricate processes that shape the respiratory system by exploring the mechanisms underlying the formation of a tracheal bronchus. This line of inquiry enhances our understanding of developmental biology and holds promise for advancing clinical practice related to the diagnosis and management of congenital respiratory anomalies.

Identifying these anomalies before lung surgery is of utmost importance for effective management of potential complications, as they may also affect tracheal intubation procedures. A bronchus originating directly from the trachea poses additional challenges during tracheal intubation, including the risk of atelectasis or respiratory failure. Notably, a right tracheal bronchus can ventilate various segments of the right upper lobe. When the right upper lobe bronchus is wholly displaced, the adjacent right middle bronchus may become stenotic, possibly due to the lack of developmental contribution from the right upper lobe (6).

Furthermore, it is worth noting that a left tracheal bronchus is often found in association with a right tracheal bronchus, resulting in the formation of bilateral tracheal bronchi. This contrasts with the more common presentation of bilateral right tracheal bronchi observed in cases of right isomerism. The presence of such anomalies illustrates the complexity of airway anatomical variations. It highlights the importance of detailed preoperative assessments to ensure optimal surgical outcomes and minimize potential risks associated with anomalies and subsequent procedures.

Imaging of developmental abnormalities of the bronchial tree is most effectively performed using multidetector computed tomography (MDCT) with multiplanar reconstruction and three-dimensional imaging. Clinically, tracheal bronchi may present with symptoms such as recurrent pneumonia, stridor, or respiratory distress in children, whereas adults typically remain asymptomatic. CT scans are adept at identifying tracheal bronchi, which typically originate from the distal trachea within 2 cm above the carina (2). Documenting the distance between the tracheal bronchus and the carina in the radiology report is essential for anesthesiologists. In some cases, the abnormally branched bronchus may be small or exhibit proximal stenosis.

The main aim of this study was to determine the prevalence and distribution of tracheobronchial anomalies identified on CT imaging and to assess their association with CHD. In addition, the study explored whether the observed patterns support the “space availability” hypothesis in explaining the embryological relationship between airway and cardiovascular development.

Moreover, this study aims to shed light on the clinical implications of a tracheal bronchus. By thoroughly examining the effects of this structural anomaly on respiratory function, airway management, and possible associated complications, the research seeks to uncover its broader clinical significance. The findings of this investigation are anticipated to provide valuable insights that can influence clinical decision-making, guide diagnostic strategies, and inform the development of tailored treatment approaches for individuals presenting with this unique anatomical variation.

By bridging the gap between basic research on the pathogenesis of tracheal bronchus and its clinical implications, this study seeks to contribute to the growing body of knowledge surrounding this intriguing anatomical anomaly. Through a multidimensional exploration of the complexities underlying a tracheal bronchus, this research aims to provide a comprehensive understanding that may ultimately enhance patient care, refine medical interventions, and advance our comprehension of this intriguing aspect of respiratory anatomy.

## Materials and methods

### Study design and patient selection

This retrospective study was conducted at the National Taiwan University Hospital (NTUH) and included patients who underwent CT imaging between December 2012 and February 2024. The study was designed to include a diverse cohort of patients with congenital heart disease as well as patients without the condition, all of whom required CT imaging to provide essential information for subsequent treatment planning.

### Inclusion criteria and enrollment

The specific inclusion criteria of the study were individuals diagnosed with a lobar or segmental tracheal bronchus anomaly, as identified on their initial CT scan. Upon identifying such anomalies, patients were enrolled in the study for comprehensive analysis of their CT findings and associated clinical records.

### CT imaging analysis and classification

(a) Imaging analysis

Two radiologists, who were also researchers, thoroughly examined the CT scans of patients with congenital heart disease to identify tracheal bronchus anatomy. This approach enabled the identification and categorization of anatomical variations of the tracheal bronchus.

(b) Medical record review

Simultaneously, we reviewed the medical records of enrolled patients to extract key demographic details, including age and sex. This data served as a contextual foundation for assessing the CT findings.

(c) Diagnostic criteria for a tracheal bronchus

We established the diagnosis of a tracheal bronchus by identifying abnormal features on cross-sectional CT images. Our detailed review focused on detecting deviations from normal bronchial anatomy, specifically examining the origin, path, and branching patterns of the tracheal bronchus. To ensure consistency and precision, we applied standardized diagnostic criteria in accordance with recognized guidelines to evaluate all cases.

### Ethical considerations

This retrospective study was anonymized at the time of data collection and was conducted in accordance with the ethical standards of NTUH.

### Data analysis

The collected data were statistically analyzed using the Statistical Package for the Social Sciences (SPSS). Descriptive statistics, including frequencies, percentages, means, and standard deviations, were used to characterize the patient population and summarize the CT findings. For group comparisons, categorical variables were analyzed using the chi-square test, with Fisher's exact test applied when expected cell counts were less than five. Continuous variables, such as age, were compared using the independent-samples *t*-test, as the data were confirmed to be normally distributed. A *p*-value of <0.05 was considered statistically significant. Inferential analyses were also conducted to explore potential associations between tracheobronchial anomalies and clinical outcomes, adjusting for relevant confounding variables.

In summary, this study aims to provide a thorough and systematic evaluation of tracheobronchial anomalies using a well-defined patient cohort, rigorous classification of CT images, and a holistic review of clinical data to contribute valuable insights into the diagnosis and implications of this condition.

## Results

### Patient characteristics in tracheobronchial anomalies with congenital heart disease

From December 2012 to February 2024, 356 patients were diagnosed with tracheobronchial anomalies. Among them, 223 patients had concurrent CHD, including 115 women (51.6%), with a median age of 4.6 years (range, 0–49 years). The remaining 133 patients had no cardiac anomalies, including 61 women (45.9%), with a median age of 37.6 years (range, 0–96 years). The mean age of CHD patients with tracheobronchial anomalies was 4.6 years, markedly lower than the mean age of 37.6 years in patients without cardiac anomalies. This difference reflects the tendency for tracheobronchial anomalies associated with CHD to be detected early in life, often during evaluation or surgical planning for congenital cardiac defects, whereas isolated tracheobronchial variants are more frequently incidental findings in adults. Detailed baseline characteristics are summarized in Table 1.

**Table 1:** Baseline characteristics of patients with tracheobronchial anomalies.

Characteristics	Tracheobronchus		
	Number of patients (*n*)	Gender (female) (%)	Age (range) (years)
Lobar	287	141 (49%)	17.4 (0–86)
Segmental	69	35 (51%)	14.6 (0–96)
CHD cases	223	115 (51.6%)	4.6 (0–49)
No heart anomalies	133	61 (45.9%)	37.6 (0–96)

### Associated anomalies in congenital heart disease with tracheobronchial anomalies

When comparing associated anomalies, distinct differences emerged between the CHD and non-CHD groups. A right-sided aortic arch was present in 27 patients (12.1%) among those with CHD. In contrast, only one patient in the non-CHD group had this anomaly. A right-sided aortic arch was significantly more common in patients with tracheobronchial anomalies and CHD than in those with normal hearts (*p* < 0.005). Pulmonary atresia was observed in 14 patients (6.3%) in our study. This finding demonstrated statistical significance (*p* < 0.05) in cases of tracheobronchial anomalies.

Although persistent left superior vena cava (PLSVC) is a rare vascular anomaly, it is the most common thoracic venous anomaly. Typically asymptomatic and incidentally detected, PLSVC was found in 71 patients (30.5%) with tracheobronchial anomalies in our study. Its prevalence was significantly higher in patients with CHD than in those without CHD (*p* < 0.001).

Tracheal stenosis was observed in 18 patients, both with and without CHD, with 16 patients (7.2%) with CHD. This condition was significantly more common in patients with CHD than in those without CHD (*p* < 0.05).

Among patients with normal hearts, only one exhibited a coronary artery (CA) anomaly, specifically in the context of Kawasaki disease involving aneurysms of the left anterior descending (LAD) and mid-right coronary artery (RCA). In contrast, 31 patients (13.9%) in the CHD group had coronary artery anomalies, representing a significantly higher occurrence than in patients with normal heart function (*p* < 0.001). The detailed distribution of associated anomalies is summarized in Table 2.

**Table 2:** Distribution of associated anomalies in patients with tracheobronchial anomalies.

Associated anomalies	Tracheobronchus				
	CHD cases		No heart anomalies		*p*-value
	*n*	(%)	*n*	(%)	
Right-sided arch	27	12.1%	1	0.8%	<0.005
Pulmonary atresia	14	6.3%	0	0.0%	<0.05
PLSVC	71	30.5%	7	4.5%	<0.001
Tracheal stenosis	16	7.2%	2	1.5%	<0.05
Coronary artery anomalies	31	13.9%	1	0.8%	<0.001

CHD, congenital heart disease; PLSVC, persistent left superior vena cava.

### Analysis between lobar and segmental types of tracheobronchus

From Table 3, further stratification based on the type of tracheobronchial anomaly revealed that the lobar type (*n* = 287) was more common than the segmental type (*n* = 69). However, the proportion of CHD was slightly higher in the segmental type (72%) than that in the lobar type (67%). This tendency suggests that a segmental tracheobronchus, which reflects a deeper deviation in bronchial branching, may arise in conjunction with more complex cardiac malformations. Across both groups, the most frequent cardiac diagnoses included tetralogy of Fallot, pulmonary atresia, and other complex cyanotic CHDs, supporting the notion that abnormal bronchial branching often accompanies severe defects in cardiopulmonary morphogenesis.

**Table 3:** Comparison of demographic, cardiac, and associated anomalies between lobar and segmental types of tracheobronchus.

Characteristics	Tracheobronchus			
	Lobar		Segmental	
Number of patients	287		69	
Female	141 (49%)		35 (51%)	
Age (years)	17.4 (0–86)		14.6 (0–96)	
Main diagnosis				
TOF	67	23%	22	32%
DORV	12	4%	6	9%
TGA	5	2%	0	0%
Simple VSD	36	13%	7	10%
Simple ASD	6	2%	1	1%
ECD	6	2%	0	0%
Complex CHD	61	21%	14	20%
All CHDs	193	67%	50	72%
Normal heart	94	33%	19	28%
Associated anomalies				
Right-sided arch	23	8%	4	6%
Pulmonary atresia	29	10%	8	12%
PLSVC	61	21%	17	25%
Tracheal stenosis	13	5%	5	7%
Coronary artery anomalies	26	9%	5	7%

CHD, congenital heart disease; TOF, tetralogy of Fallot; DORV, double outlet right ventricle; TGA, transposition of great arteries; ECD, endocardial cushion defect; VSD, ventricular septal defect; ASD, atrial septal defect; PLSVC, persistent left superior vena cava.

The statistical analyses supported these clinical observations. Chi-square tests demonstrated significant associations between tracheobronchial anomalies and a right-sided aortic arch (*χ*^2^ = 14.83, *p* < 0.005), pulmonary atresia (*χ*^2^ = 5.50, *p* < 0.05), PLSVC (*χ*^2^ = 31.07, *p* < 0.001), tracheal stenosis (*χ*^2^ = 5.58, *p* < 0.05), and coronary artery anomalies (*χ*^2^ = 62.47, *p* < 0.001). The magnitude of association was strongest for PLSVC and coronary anomalies, both of which likely share common embryological origins with the tracheobronchial tree.

Taken together, these results illustrate a clear developmental and clinical pattern. Patients with CHD not only exhibit a higher prevalence of tracheobronchial anomalies but also demonstrate a clustering of vascular abnormalities, most notably PLSVC and coronary variations, likely reflecting shared embryonic pathways (Table 4). The presence of tracheobronchial anomalies in individuals with structurally normal hearts appears to be sporadic and clinically less consequential. In contrast, their occurrence in patients with CHD reflects a more intricate interplay in cardiopulmonary morphogenesis. This finding supports the “space availability” hypothesis, which is discussed in greater detail in the following section, which proposes that aberrant bronchial branching during early embryogenesis may modify mediastinal spatial relationships, thereby influencing the concurrent development of adjacent vascular structures.

**Table 4:** Chi-square test of independence.

Chi-square independence value (*χ*^2^)	Items	*p*-value
14.82537832	R arch	<0.005
5.506933227	Pul atresia	<0.05
31.07329757	PLSVC	<0.001
5.581568661	Tracheal stenosis	<0.05
62.46750912	Coronary artery anomalies	<0.001

The findings of this study demonstrate that tracheobronchial anomalies occur significantly more often in conjunction with congenital heart diseases than in structurally normal hearts. Recognition of these patterns is important for improving diagnostic accuracy, guiding surgical planning, and anticipating perioperative challenges in patients with CHD.

## Discussion

This study aims to comprehensively investigate tracheal bronchus anomalies and their clinical implications, shedding light on their prevalence, associated congenital heart diseases, diagnostic methods, and potential complications.

### Prevalence

The prevalence of tracheal bronchus anomalies, although low at 0.1%–2%, holds clinical significance, especially in patients with congenital heart disease. Based on autopsy and catheter angiographic data, the incidence of CA anomalies in the general population with structurally normal hearts is approximately 1.0% (0.3%–2.2%) (1). Another study by Wang et al. reported airway anomalies in heterotaxy syndrome. The study revealed that congenital lower airway anomalies/stenosis are common in heterotaxy syndrome (27.4%) and may increase the duration of ventilator care during initial palliative cardiac surgery (7). This study highlights that the association between tracheal bronchus and congenital heart diseases suggests a potential link in their embryological origins or underlying pathophysiology.

### Embryological basis and pathogenesis

The embryological origins of tracheal bronchus anomalies remain a subject of interest and speculation. Bremer's hypothesis, which proposes a failure of regression of tracheal buds, offers a compelling explanation and is supported by experimental evidence suggesting the influence of external factors on embryonic development (4). Further research into the molecular mechanisms and genetic determinants underlying tracheal bronchus formation may provide valuable insights into its pathogenesis and potential preventive strategies.

Several hypotheses have been proposed to explain the development of PLSVC. One of these is the “low left atrial pressure” theory, which suggests that certain anomalies may cause reduced left atrial pressure and insufficient development of the left atrium. Consequently, the left common cardinal vein and the caudal part of the left superior cardinal vein fail to regress, leading to the development of PLSVC. According to the “obstructive theory” hypothesis, the presence of PLSVC may enlarge the coronary size and contribute to the development of a left-sided obstructive lesion due to space restriction (8). However, our study found that seven patients with tracheobronchial anomalies had PLSVC despite having normal hearts.

As the lung buds repeatedly divide, they form the bronchial tree. Signals modulated by the surrounding mesenchyme guide the branching pattern of lung buds. Chen et al. hypothesized the “space availability” theory to explain the relationship between a tracheal bronchus and a left pulmonary artery (LPA) sling. Patients with an LPA sling likely experience early branching of the right upper bronchus during embryogenesis, leaving a wider space around the lower developing primitive trachea. Such development leaves more space around the primitive carina, thereby increasing the likelihood that the left postbranchial pulmonary vessel will approach the right ventral sixth branchial arch. This also correlates with PLSVC due to increased space on the left side. In our study, 68 patients with PLSVC and congenital heart disease had tracheobronchial anomalies. The hypothesis of “space availability” suggests that the right tracheobronchus leaves a wider space on the left side, making PLSVC occurrence more common. However, during embryogenesis *in utero*, the left common cardinal vein and the caudal part of the left superior cardinal vein regress during the 8th week of development (9).

### Coronary artery theory

The coexistence of tracheobronchial anomalies and coronary artery abnormalities may reflect shared embryologic origins. During early cardiopulmonary development, both coronary and bronchial structures arise from the same mesenchymal region of the primitive foregut and aortic sac. Spatial competition within this confined mediastinal region may result in simultaneous aberrations in vascular and airway branching. Abnormal regression or persistence of primitive coronary channels may therefore occur alongside atypical bronchial budding when local mesenchymal signaling is disrupted.

This embryologic interaction supports the “space availability” theory, suggesting that mechanical or spatial constraints during organogenesis can contribute to parallel anomalies of the coronary and tracheobronchial systems. The close temporal overlap in the development of the coronary arteries (from epicardial and endothelial buds around the 5th week) and the bronchial tree (from the foregut diverticulum around the 4th week) may predispose these structures to mutual morphogenetic influence.

Studies by Chao et al. (10) and Song et al. (11) further support this concept, reporting that tracheobronchial anomalies often coexist with vascular abnormalities, including pulmonary artery sling and coronary malformations, likely reflecting a shared mesenchymal developmental domain. The findings of the present study, where 37.7% of CHD patients with tracheobronchial anomalies also exhibited coronary artery variations, strengthen the evidence that these two systems may be developmentally interdependent rather than coincidentally associated. Our data, showing a high prevalence of PLSVC (30.5%) among CHD patients, further support the “space availability” hypothesis, which proposes that altered mediastinal geometry during embryogenesis influences both bronchial and vascular morphogenesis.

### Clinical implications and management

The clinical implications of tracheal bronchus anomalies extend beyond anatomical variations, affecting respiratory function, airway management, and surgical outcomes. Early detection and accurate diagnosis are crucial for optimizing patient care and minimizing associated complications. The utilization of MDCT and advanced imaging techniques facilitates precise visualization of bronchial tree anomalies, enabling clinicians to tailor treatment strategies accordingly.

### Association with congenital heart disease

The significant association between tracheal bronchi and congenital heart disease, particularly a right-sided aortic arch and pulmonary atresia, highlights the need for interdisciplinary collaboration in managing these complex conditions. Our study identified tracheal stenosis in 18 patients, including 16 (7.2%) with CHD. This condition was significantly more common in patients with CHD than in those without CHD (*p* < 0.05). These findings are consistent with a retrospective study by Wang et al., which reported airway anomalies in 31% of patients with CHD. The study also noted that such anomalies were associated with a significantly longer hospital stay, even after adjustment for other risk factors for severe illness. The presence of airway anomalies can complicate the surgical management of CHD, potentially increasing the risk of bronchiolitis and related hospital admissions (12). Understanding the coexistence of these anomalies can guide preoperative assessments, surgical planning, and postoperative care, ultimately improving patient outcomes.

The notable age difference between patients with and without congenital heart disease (mean 4.6 vs. 37.6  ears) likely reflects the distinct clinical indications for CT imaging in each group. In our cohort, patients with CHD predominantly underwent CT evaluation during infancy or early childhood, often as part of preoperative or follow-up cardiac assessment, whereas individuals without CHD were mostly adults who underwent CT for unrelated respiratory or incidental findings. This discrepancy introduces an inherent selection bias and potential confounding effect, as the timing and purpose of imaging influence the likelihood of detecting tracheobronchial anomalies. Moreover, developmental factors and differential survival across age groups may affect the apparent prevalence of tracheobronchial variants. Therefore, the observed associations between CHD and tracheobronchial anomalies should be interpreted with caution, acknowledging that they may partly reflect age-related sampling differences rather than true biological causality.

### Study strengths and limitations

This retrospective design enabled analysis of a large cohort over 12 years; however, several limitations warrant consideration. First, there was a significant age difference between the CHD and non-CHD groups (4.6 vs. 37.6 years), primarily due to differing CT indications—presurgical evaluation in pediatric CHD cases vs. adult diagnostic imaging for non-cardiac conditions. This introduces a potential confounding effect, as younger patients are more likely to undergo systematic evaluation of congenital anomalies. Second, the single-center, retrospective nature of the study may limit generalizability. Finally, selection bias cannot be excluded, as only patients who underwent CT imaging were included. Despite these limitations, the consistent imaging protocol and detailed anomaly classification strengthen the validity of our findings.

### Future directions

Although this study demonstrated statistically significant associations between tracheobronchial anomalies and specific cardiovascular defects, the analyses were primarily descriptive and exploratory. Because the CHD group consisted predominantly of pediatric patients, while the non-CHD group included mostly adults, age was regarded as an inherent characteristic rather than a confounding variable. The observed associations therefore reflect distinct developmental pathways rather than demographic differences. Nevertheless, future studies with larger and more balanced cohorts are encouraged to perform multivariate analyses to validate these findings.

Future research should aim to elucidate the mechanisms underlying the link between tracheobronchial anomalies and congenital cardiovascular malformations. Specifically, multi-institutional studies integrating embryologic imaging, genetic sequencing, and morphogenetic modeling may help identify the signaling pathways responsible for concurrent airway and vascular maldevelopment.

Advanced imaging technologies, such as high-resolution fetal MRI and 4D flow CT, may allow dynamic visualization of cardiopulmonary development, enabling real-time assessment of spatial constraints that support the “space availability” hypothesis. Prospective studies employing these modalities could help clarify whether tracheal bronchus formation precedes, follows, or coincides with vascular remodeling during embryogenesis.

## Conclusion

Tracheobronchial anomalies show a strong association with congenital heart disease, particularly with persistent left superior vena cava and coronary artery anomalies, supporting the hypothesis of a shared embryologic origin between the airway and cardiovascular systems. Disruptions in early cardiopulmonary morphogenesis may underlie their concurrent development. This study enhances understanding of the epidemiology and morphologic spectrum of these anomalies while underscoring their clinical importance in patients with CHD. The integration of high-resolution CT imaging with clinical data allows for early recognition and precise characterization, thereby aiding surgical and anesthetic planning. By linking embryologic theory with clinical evidence, this study provides a unified developmental perspective on congenital thoracic anomalies and highlights the need for multidisciplinary collaboration to improve diagnostic accuracy and patient outcomes.

## Data Availability

The raw data supporting the conclusions of this article will be made available by the authors, without undue reservation.

## References

[1] Chassagnon G Morel B Carpentier E Ducou Le Pointe H Sirinelli D. Tracheobronchial branching abnormalities: lobe-based classification scheme. Radiogr Rev Publ Radiol Soc N Am Inc. (2016) 36(2):358–73. 10.1148/rg.2016150115 26824513

[2] Ulusoy M Kivrak AS Uysal II Karabulut AK Paksoy Y Fazliogullari Z. Developmental anomalies of bronchial tree: a multidetector computerized tomography study. Int J Morphol. (2013) 31(3):1049–55. 10.4067/S0717-95022013000300044

[3] Chassagnon G Lefort B Meot M Carpentier E Sirinelli D Chantepie A Association between tetralogy of Fallot and tracheobronchial branching abnormalities: a new clue for pathogenesis? J Am Heart Assoc. (2018) 7(1):e006921. 10.1161/JAHA.117.006921 PMC5778959 29288155

[4] Kim H Kim YT Jou SS Lee WH. True tracheal bronchus: classification and anatomical relationship on multi-detector computed tomography. J Korean Soc Radiol. (2017) 76(4):264–72. 10.3348/jksr.2017.76.4.264

[5] Alescio T Cassini A. Induction *in vitro* of tracheal buds by pulmonary mesenchyme grafted on tracheal epithelium. J Exp Zool. (1962) 150(2):83–94. 10.1002/jez.1401500202 14011906

[6] Wise-Faberowski L Irvin M Sidell DR Rajashekara S Asija R Chan FP Assessment of airway abnormalities in patients with tetralogy of Fallot, pulmonary atresia, and major aortopulmonary collaterals. Cardiol Young. (2019) 29(5):610–4. 10.1017/S1047951119000301 31044684

[7] Wang CC Wu MH Wu ET Lu F Chen SJ. Clinical implications of airway anomalies and stenosis in patients with heterotaxy syndrome. Pediatr Pulmonol. (2022) 57(9):2074–81. 10.1002/ppul.25981 35582940

[8] Azizova A Onder O Arslan S Ardali S Hazirolan T. Persistent left superior vena cava: clinical importance and differential diagnoses. Insights Imaging. (2020) 11(1):110. 10.1186/s13244-020-00906-2 33057803 PMC7561662

[9] Chen SJ Lee WJ Lin MT Wang JK Chang CI Chiu IS Left pulmonary artery sling complex: computed tomography and hypothesis of embryogenesis. Ann Thorac Surg. (2007) 84(5):1645–50. 10.1016/j.athoracsur.2007.05.094 17954077

[10] Chao YC Peng CC Lee KS Lin SM Chen MR. The association of congenital tracheobronchial stenosis and cardiovascular anomalies. Int J Pediatr Otorhinolaryngol. (2016) 83:1–6. 10.1016/j.ijporl.2016.01.024 26968043

[11] Song X Lu Z Zhu L Du X Wang S Xu Z. Morphologic analysis of congenital heart disease with anomalous tracheobronchial arborization. Ann Thorac Surg. (2020) 110(4):1387–95. 10.1016/j.athoracsur.2020.01.027 32114043

[12] Wang PY Tseng WC Wu ET Lu FL Chen SJ Chiu SN The implications of airway anomalies on children with congenital heart disease and bronchiolitis. Pediatr Pulmonol. (2023) 58(4):1194–200. 10.1002/ppul.26320 36650613

